# Compositional Changes of B and T Cell Subtypes during Fingolimod Treatment in Multiple Sclerosis Patients: A 12-Month Follow-Up Study

**DOI:** 10.1371/journal.pone.0111115

**Published:** 2014-10-31

**Authors:** Nele Claes, Tessa Dhaeze, Judith Fraussen, Bieke Broux, Bart Van Wijmeersch, Piet Stinissen, Raymond Hupperts, Niels Hellings, Veerle Somers

**Affiliations:** 1 Hasselt University, Biomedical Research Institute and Transnationale Universiteit Limburg, School of Life Sciences, Diepenbeek, Belgium; 2 Rehabilitation & MS-Center, Overpelt, Belgium; 3 Department of Neuroscience, School of Mental Health and Neuroscience, Maastricht University, Maastricht, The Netherlands; 4 Department of Neurology, Orbis Medical Center, Sittard, The Netherlands; University Hospital Basel, Switzerland

## Abstract

**Background and objective:**

The long term effects of fingolimod, an oral treatment for relapsing-remitting (RR) multiple sclerosis (MS), on blood circulating B and T cell subtypes in MS patients are not completely understood. This study describes for the first time the longitudinal effects of fingolimod treatment on B and T cell subtypes. Furthermore, expression of surface molecules involved in antigen presentation and costimulation during fingolimod treatment are assessed in MS patients in a 12 month follow-up study.

**Methods:**

Using flow cytometry, B and T cell subtypes, and their expression of antigen presentation, costimulation and migration markers were measured during a 12 month follow-up in the peripheral blood of MS patients. Data of fingolimod-treated MS patients (n = 49) were compared to those from treatment-naive (n = 47) and interferon-treated (n = 27) MS patients.

**Results:**

In the B cell population, we observed a decrease in the proportion of non class-switched and class-switched memory B cells (p<0.001), both implicated in MS pathogenesis, while the proportion of naive B cells was increased during fingolimod treatment in the peripheral blood (PB) of MS patients (p<0.05). The remaining T cell population, in contrast, showed elevated proportions of memory conventional and regulatory T cells (p<0.01) and declined proportions of naive conventional and regulatory cells (p<0.05). These naive T cell subtypes are main drivers of MS pathogenesis. B cell expression of CD80 and CD86 and programmed death (PD) -1 expression on circulating follicular helper T cells was increased during fingolimod follow-up (p<0.05) pointing to a potentially compensatory mechanism of the remaining circulating lymphocyte subtypes that could provide additional help during normal immune responses.

**Conclusions:**

MS patients treated with fingolimod showed a change in PB lymphocyte subtype proportions and expression of functional molecules on T and B cells, suggesting an association with the therapeutic efficacy of fingolimod.

## Introduction

A complex interplay between T and B cells drives the disease course of multiple sclerosis (MS). Thereby, non class-switched (CD19^+^IgD^+^CD27^+^) and class-switched (CD19^+^IgD^-^CD27^+^) memory B cells are generally considered to be the main pathogenic B cell subtypes, whereas, conventional (autoreactive) T cells (CD4^+^CD25^-^CD127^+^) can drive the disease and regulatory T cells (CD4^+^CD25^hi^CD127^lo^) control immune homeostasis [1]–[3]. Both within the conventional and regulatory T cell populations, naive (CD45RA^+^CD45RO^-^) and memory (CD45RA^-^CD45RO^+^) subtypes can be discriminated. The role of other peripheral blood (PB) immune cells in MS pathogenesis, such as naive B cells (CD19^+^IgD^+^CD27^-^), double negative B cells (CD19^+^IgD^-^CD27^-^) and follicular helper T cells (TFH; CD4^+^CD25^-^CD127^+^CXCR5^+^PD-1^+^), is still unclear. B and T cells interact via surface molecules e.g. human leukocyte antigen (HLA)-DR/DP/DQ, CD80 and CD86 on B cells and programmed death (PD) -1 on T cells. Furthermore, migration of B and T cells is partly mediated via chemokine (C-X-C motif) receptor 5 (CXCR5) [4],

Fingolimod is the FDA approved oral treatment for MS and has shown efficacy in relapsing remitting (RR) MS [5]–[8]. Fingolimod is an immunomodulator that interferes with the signaling of the sphingosine-1-phospate receptor 1 (S1PR1), present on lymphocytes, and causes the internalization and degradation of this receptor [9]. Consequently lymphocytes cannot exit the lymph nodes into the circulation, leading to the entrapment of lymphocytes in lymphatic systems, causing lymphopenia in peripheral blood (PB) of treated patients, thereby reducing the number of inflammatory cells migrating to the central nervous system (CNS) [9]–[12].

Limited information is available concerning the effects of fingolimod on different T and B cell subtypes and on the interplay between these lymphocyte populations in the PB of MS patients [13]–[15]. To understand the longitudinal immunological effects of fingolimod treatment, we investigated the effect of this treatment on B and T cell subtypes and antigen presentation, costimulation and migration molecules expressed on these cells in PB of MS patients in a 12 months follow-up study.

## Materials and Methods

### Study population

PB was collected from MS patients in both the Orbis Medical Center (Sittard, the Netherlands) and Rehabilitation and MS-center (Overpelt, Belgium). For PB collection in the Orbis Medical Center, written informed consent was obtained from all participants after approval by the Medical ethical Committee Atrium-Orbis-Zuyd (12-N-56). Furthermore, PB was collected by the Rehabilitation and MS-center in Overpelt after written informed consent from all participants and approval by the UZ Leuven and Hasselt University Commissions of Medical Ethics (S54362 and S54363). A total of 123 MS patients were involved in the study, including 47 treatment-naive MS patients, 27 MS patients on interferon-β (IFN-β) treatment (together referred to as controls) and 49 MS patients on fingolimod treatment (0.5 mg/day). All MS patients were diagnosed according to the revised McDonald criteria [16]. Treatment-naive MS patients never received any MS related treatment. PB of the fingolimod-treated group was collected after wash-out of the previous treatment (minimally 2 months) and before the first dose of fingolimod (baseline). MS patients were then followed over time: PB was collected after 1 month (1 m), 3 months (3 m) and every 3 consecutive months of treatment for a period of up to 12 months (6 m, 9 m, 12 m). Clinical non-responders to fingolimod treatment were characterized by an increase in EDSS score, a relapse or a new magnetic resonance imaging (MRI) lesion after a minimum of 3 months of fingolimod treatment and were excluded from the study.

### Flow cytometry

PB was collected in heparin-coated tubes (Venosafe plastic tubes, Terumo Europe N.V., Leuven, Belgium) and PB mononuclear cells (PBMC) were isolated using high density centrifugation (*Lympholyte*; *Cedarlane* Laboratories, SanBio B.V., Uden, the Netherlands). PBMC (0.5×10^6^ cells) were stained using anti-human CD19 PerCP-Cy5.5 and CD4 APC to discriminate between B and T cells, respectively (BD Biosciences, Erembodegem, Belgium). B cell subpopulations and surface molecules were defined using following anti-human antibodies: IgD APC-Cy7, CD27 PE-Cy7, HLA-DR/DP/DQ (major histocompatibility complex (MHC)-II) FITC, CD80 PE and CD86 PE-CF594 (all from BD Biosciences, Erembodegem, Belgium). Following anti-human monoclonal antibodies were used for T cell analysis: CD45RA APC-H7, CD45RO PE-CF594, CXCR5 Alexa Fluor 488 and PD-1 PE-Cy7 (all from BD Biosciences, Erembodegem, Belgium), CD25 PerCP-Cy5.5 and CD127 PE (eBioscience, San Diego, USA). Following isotype controls were used: mouse IgG1 PerCP-Cy5.5, IgG1 PE, IgG1 Pe-Cy7, IgG2aκ PE-CF594, IgG2bκ APC-H7, IgG1 APC, IgG2aκ FITC, IgG1κ PE-CF594, IgG1 Pe-Cy7, IgG2aκ APC-H7 and rat IgG2b Alexa Fluor 488 (all from BD Biosciences, Erembodegem, Belgium). All flow cytometric analyses were performed on a FACSAriaII flow cytometer and analyzed with FACS Diva software (BD Biosciences).

### Statistical analysis

Data analysis was performed using Prism software version 5.01 (graphpad) and SAS 9.3 software. Appropriate One-way ANOVA analysis was used with Dunn's multiple comparison post-hoc test for comparison of treatment controls and baseline fingolimod-treated patients after normality check (Kolmogorov-Smirnov). A mixed model was used for data analysis of treatment follow-up compared to baseline fingolimod. A p-value of <0.05 was considered statistically significant.

## Results

### Reduction of total PB lymphocyte, B and T cell counts after fingolimod treatment

**Figure 1:**
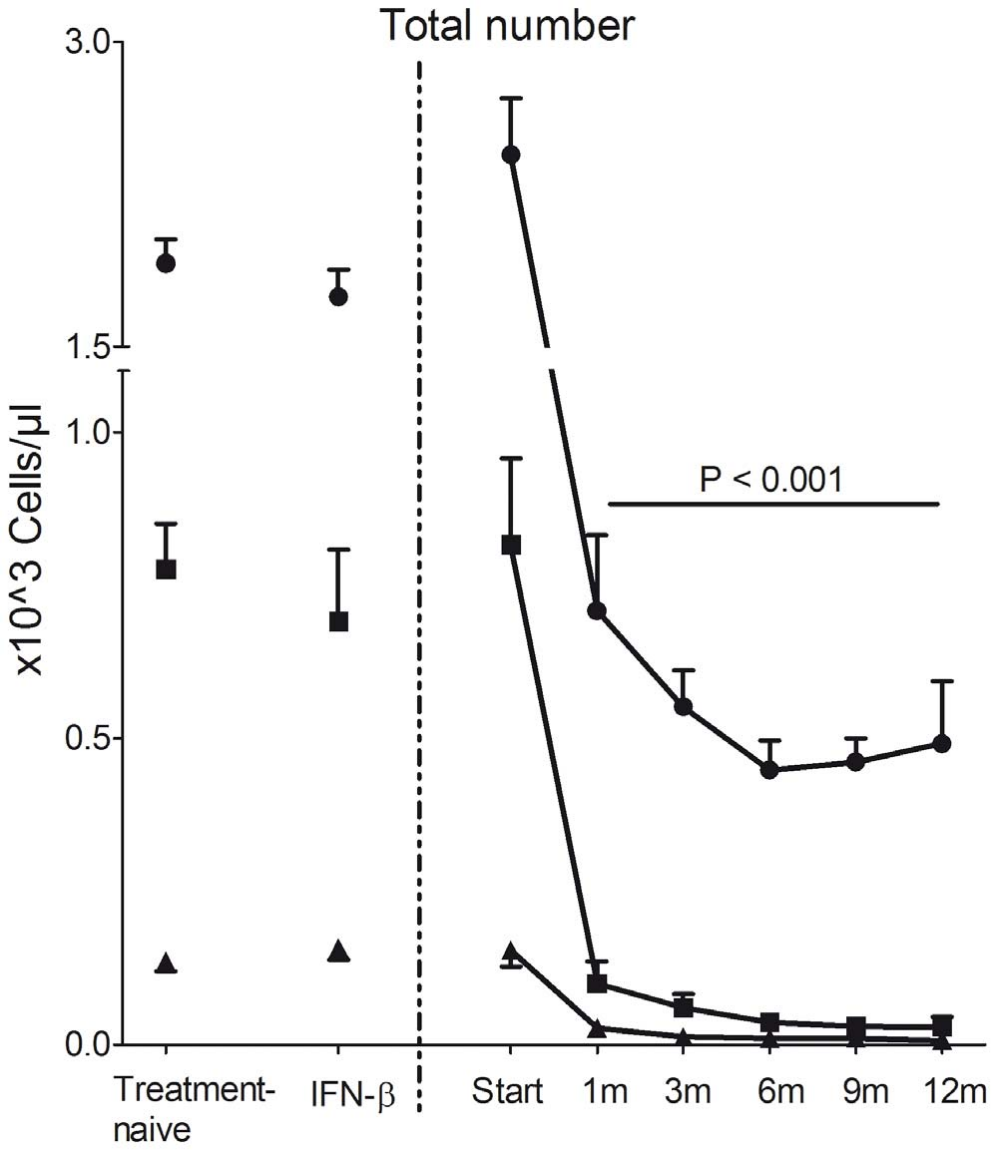
Total number of lymphocytes, CD4^+^ T cells and CD19^+^ B cells in the PB. Total number (×10^3^ cells/µl blood) of lymphocytes, T cells and B cells in treatment-naive, IFN-β treated MS patients at baseline and fingolimod-treated MS patients during 12 months follow-up. Mean and standard error of the mean are presented. • lymphocytes; ▪ CD4^+^ T cells; ▴ CD19^+^ B cells.

In total, PB of 49 fingolimod-treated MS patients was collected at different time points up to 12 months of treatment. The cohort of fingolimod-treated MS patients was compared at baseline with 47 treatment-naive and 27 IFN-β treated MS patients (together referred to as controls). Fingolimod-treated MS patients at baseline and controls were comparable in terms of age, gender distribution and median EDSS score (table 1). Furthermore, no significant difference was observed in numbers of total lymphocytes, B cells or T cells (figure 1) between baseline fingolimod treatment and controls. For the MS patients receiving fingolimod treatment, pretreatment (baseline) values were used as reference to assess the effects of treatment. Five of 49 fingolimod-treated MS patients did not finish the study due to side effects caused by the treatment. Seven MS patients were excluded from the study as clinical non-responders, although no differences in T and B cell subtype proportions between non-responders and responders were found (data not shown).

**Table 1:** Study population.

			Gender	Classification^b^		EDSS^c^ (range)
		Age^a^ (range)	F/M	RR	CP	
**Total (n = 123)**		44 (17–79)	90/33	92	23	2.5 (0.0–7.0)
**Treatment naive (n = 47)**		48 (17–79)	33/14	29	12	2.5 (0.0–7.0)
**Interferon (n = 27)**		42 (17–66)	19/8	22	5	2.5 (1.0–6.5)
**Fingolimod (n = 49)**		44 (18–69)	38/11	43	6	2.5 (0.0–6.5)
Non-responders (n = 7)		49 (34–54)	5/2	4	3	4.0 (1.0–6.5)
Drop outs (n = 5)		41 (32–56)	5/0	5	0	2.0 (1.5–6.0)
0 m (n = 28)		43 (18–67)	21/7	25	3	2.5 (0.0–6.0)
1 m (n = 24)		41(18–67)	18/6	22	2	2.5 (0.0–6.0)
3 m (n = 29)		43 (18–67)	22/7	26	3	2.5 (0.0–6.0)
6 m (n = 26)		43 (18–69)	20/6	24	2	2.5 (0.0–6.0)
9 m (n = 27)		45 (18–69)	23/4	24	3	2.5 (0.0–6.0)
12 m (n = 13)		45 (29–69)	11/2	12	1	2.5 (0.0–5.0)

a. Mean age in years.

b. For 6 treatment-naive patients, MS type was not specified.

c. Median EDSS score; this information was not available for 7 treatment-naive patients and 6 IFN-β-treated patients.

Abbreviations: F =  female; M =  male; RR =  relapsing-remitting MS; CP =  chronic progressive MS; EDSS =  expanded disability status scale, m =  month.

Total lymphocyte numbers were decreased after 1 month (1 m) of fingolimod treatment compared with baseline and controls for the total duration of the study (12 m) (p<0.001; figure 1). Furthermore, total CD19^+^ B cell and CD4^+^ T cell numbers were decreased at 1 m and reached a steady state at 3 m (p<0.001; figure 1). Similar results were observed for the percentage of CD19^+^ and CD4^+^ cells within the lymphocyte population (p<0.001; table S1).

### Fingolimod affects B cell subtype distribution in the PB of MS patients

During immune responses, B cells produce antibodies after maturation into plasma cells, function as antigen presenting cells, provide costimulation for T cells and play a role in immune memory. In MS, memory B cells and plasma cells may contribute to the pathogenesis by production of autoantibodies and cytokines [17].

**Figure 2:**
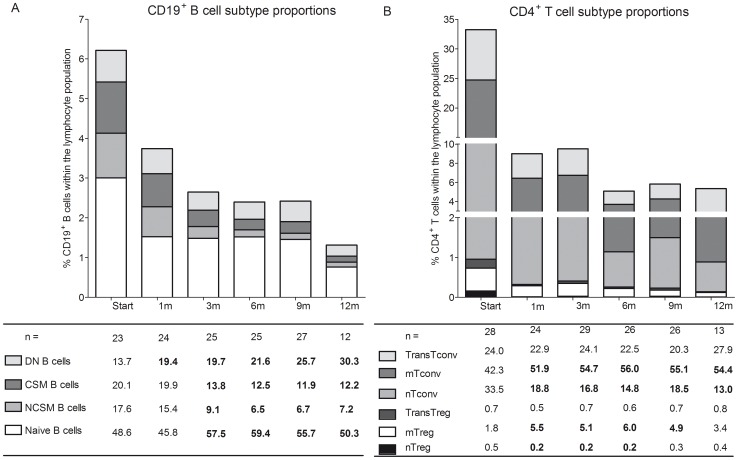
Proportional B cell and T cell subtype changes in MS patients during fingolimod treatment. (A) CD19^+^ B cell subtype proportion and (B) CD4^+^ T cell subtype proportion within the PB of treatment-naive, IFN-β and fingolimod-treated MS patients. Results are presented as relative values within the CD19^+^ B cell or CD4^+^ T cell population. Subtype proportions were calculated as follows: (% subtype/100)×% CD19^+^ or CD4^+^ within the total lymphocyte population. Statistically significant differences compared to 0 m are shown in bold. For B cells: naive B cells; NCSM B cells  =  non class-switched memory B cells; CSM B cells  =  class-switched memory B cells and DN B cells  =  double negative B cells. For T cells: nTreg  =  naive Treg; mTreg  =  memory Treg; TransTreg  =  transitional Treg; nTconv  =  naive Tconv; mTconv  =  memory Tconv; TransTconv  =  transitional Tconv.

Although B cell numbers were reduced in the PB after fingolimod treatment, we investigated the effects of fingolimod treatment on the remaining B cell population in the PB of treated patients. Both non class-switched (CD19^+^IgD^+^CD27^+^) and class-switched memory B cells (CD19^+^IgD^-^CD27^+^) were significantly decreased in the peripheral B cell population from 3 m until end of follow-up (p<0.001; figure 2A, table S1, figure S1). In contrast, the percentage of CD19^+^IgD^-^CD27^-^ cells (double negative B cells) was significantly increased within the B cell population at 1 m up to 12 m (p<0.05 at 1 m; p<0.001 at 3–12 m; figure 2A, table S1, figure S1). Naive B cells (CD19^+^IgD^+^CD27^-^) made up about 50% of the remaining peripheral B cells and the proportion of these cells was increased after 3 m fingolimod until end of follow-up (p<0.03; figure 2A; table S1, figure S1).

Distribution of B cell subtypes at start of fingolimod treatment was the same as in treatment-naive and IFN-β-treated MS patients (table S1). In general, fingolimod treatment caused a decline in memory B cell subpopulations while naïve and double negative B cell proportions were increased in the PB of MS patients.

### Change in surface expression of molecules involved in B cell antigen presentation and costimulation under fingolimod treatment

B cells are potent antigen presenting cells via the surface molecule HLA-DR/DP/DQ (MHC-II) and are important to provide costimulation to T cells via the surface molecules CD80 and CD86 [18].

**Figure 3:**
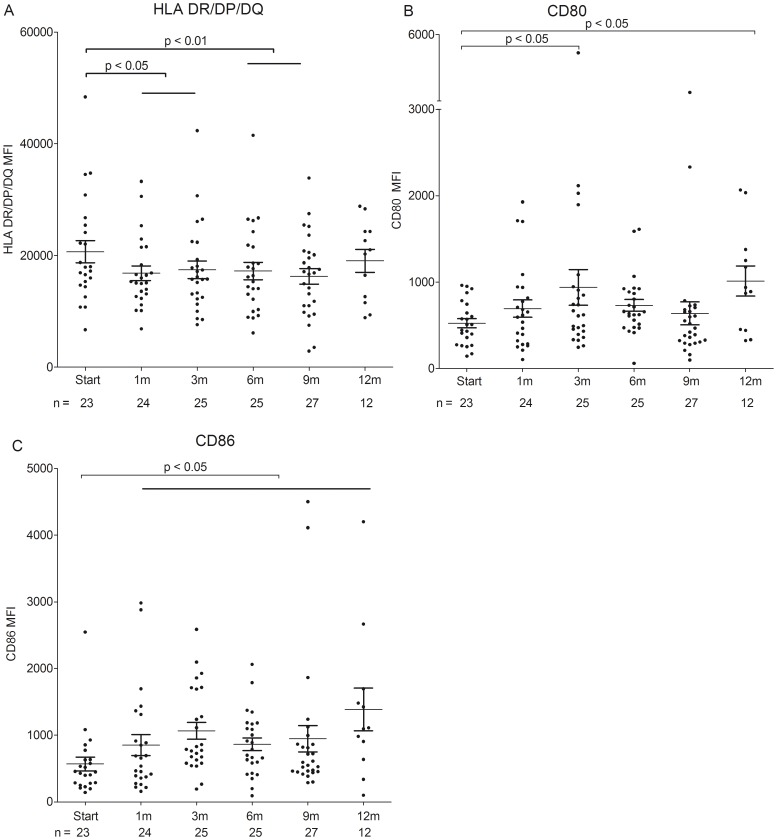
B cell expression levels of antigen presentation and costimulation molecules during fingolimod treatment. Mean fluorescence intensity (MFI) of (A) HLA-DR/DP/DQ, (B) CD80 and (C) CD86 expression within the B cell population from fingolimod-treated MS patients during follow-up.

During fingolimod treatment, both the percentage of HLA-DR/DP/DQ, CD80 and CD86 positive cells and the expression of these surface markers on CD19^+^ B cells was assessed using flow cytometric analysis. The percentage of HLA-DR/DP/DQ^+^ B cells (data not shown) and the expression of HLA-DR/DP/DQ (MFI) on B cells was significantly decreased after 3 m and 1 m of fingolimod treatment, respectively, in comparison with baseline (p<0.05; figure 3A; table S2). Fingolimod treatment resulted in an increased expression of both CD86 (after 1 m) and CD80 (after 3 m and 12 m) on B cells (MFI) compared with baseline (p<0.05; figure 3B and C; table S2). The percentages of CD80^+^ and CD86^+^ B cells remained stable during the follow-up period (table S2). Expression of antigen presentation and costimulation markers on B cells was comparable between baseline fingolimod and controls (table S2). Thus, the expression of HLA-DR/DP/DQ on PB B cells was decreased (both percentage of positive cells and MFI), while the expression of the costimulation molecules CD80 and CD86 (MFI) was increased during fingolimod treatment in MS patients.

### Fingolimod affects conventional and regulatory T cell subtype distribution in the PB of MS patients

Conventional T cells (Tconv, CD4^+^CD25^-^CD127^+^) are considered to be main players in maintaining a normal immune response and exert autoreactivity in autoimmune diseases like MS [1]. Regulatory T cells (Treg, CD4^+^CD25^hi^CD127^lo^) are essential for immune homeostasis and were shown to be functionally impaired in MS pathogenesis [1].

The longitudinal effects of fingolimod treatment on different CD4^+^ T cell subtypes including naive (CD45RA^+^CD45RO^-^), memory (CD45RA^-^CD45RO^+^) and transitional (CD45RA^+^CD45RO^+^) cells within both Tconv and Treg populations were assessed using flow cytometry. For the Tconv population, the proportion of naive cells was decreased in fingolimod-treated patients at all timepoints measured, when compared with baseline (p<0.001; figure 2B; table S1, figure S2). In contrast, a significant increase in the percentage of memory Tconv was observed after 1 m until 12 m in comparison with baseline (p<0.001; figure 2B; table S1, figure S2).

The percentage of naive cells within the Treg subtypes displayed a significant decrease after 1 m, 3 m and 6 m (p<0.05; figure 2B; table S1, figure S2) while a significant increase was observed in the proportion of memory Tregs after 1 m until 9 m (p<0.001), as observed for the Tconv population.

Interestingly, the transitional T cells (CD45RA^+^CD45RO^+^), both in the regulatory and conventional T cell population, remained stable throughout the 12 month follow-up period. Of note, baseline levels of the fingolimod-treated groups differed significantly compared with treatment-naive patients for Tconv and Treg (p<0.05; table S1). Similar changes were observed when comparing IFN-β-treated MS patients to the treatment-naive group. Together, these results show that fingolimod treatment caused a decrease in the proportions of naïve Tconv and Treg cells in the PB, together with an increase in the proportion of memory Tconv and Treg cells.

### PD-1 expression increases on circulating follicular helper T cells during fingolimod treatment

Circulating CXCR5^+^ PD-1^+^ follicular helper T cells (T_FH_) have the capacity to recirculate in secondary lymphoid organs where they can interact with B cells and influence the germinal center response [19].

**Figure 4:**
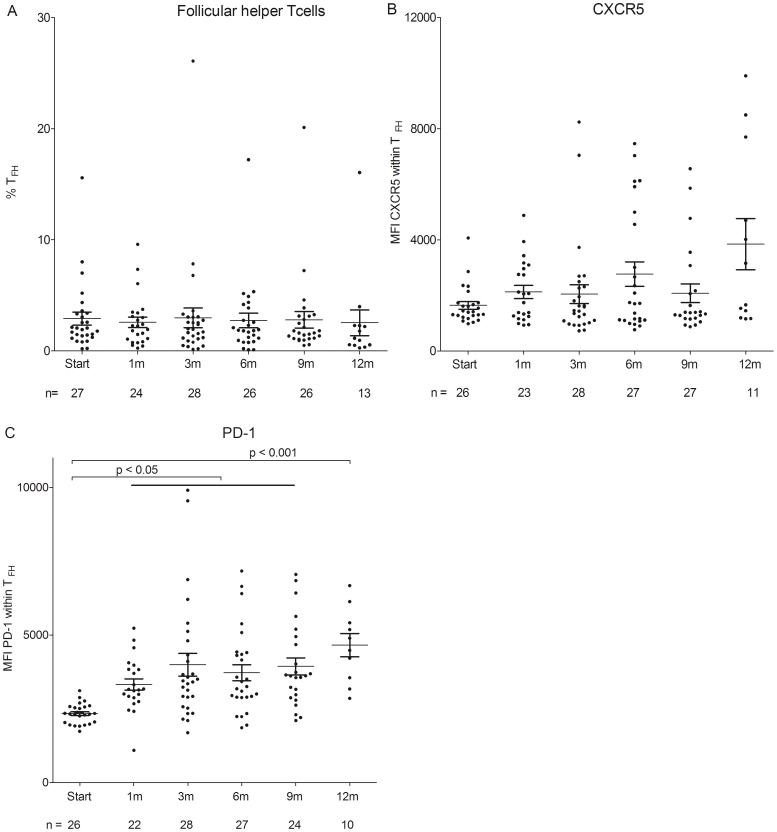
Percentage of T_FH_ and expression of CXCR5 and PD-1 during fingolimod treatment in MS patients. (A.) Percentage of PB follicular helper T cells (T_FH_) in MS patients treated with fingolimod. Data are presented as percentage within the CD4^+^ T cell population. (B.) Expression of CXCR5 and (C.) expression of PD-1 within T_FH_ cell population.

The percentage of T_FH_ (CD4^+^CD25^-^CD127^+^CXCR5^+^PD-1^+^) remained stable within the CD4^+^ population during fingolimod treatment (figure 4A, table S1).

To assess the effect of fingolimod on the expression of molecules involved in cell migration towards the germinal center and molecules involved in the functionality of the germinal center response, CXCR5 and PD-1 expression levels were determined on T_FH_ cells [19]–[22]. While the expression of CXCR5 on T_FH_ did not change in fingolimod-treated MS patients (figure 4B; table S2), a significant increase of the expression of PD-1 on PB T_FH_ cells was observed during follow-up (p<0.05; figure 4C; table S2). These results shows that the frequency of circulating T_FH_ cells that egress from the lymph nodes was not affected by treatment with fingolimod while expression of PD-1 on these T_FH_ cells in the PB of MS patients was increased.

## Discussion

In this study, we elucidate the effects of fingolimod, approved as therapy for RR-MS, on different B and T cell subtypes and expression of surface molecules involved in antigen presentation, costimulation and migration during a 12 month follow-up study. Under fingolimod treatment, the B cell subtype distribution changed, resulting in a decreased proportion of memory B cells and an increased proportion of naive and double negative B cells in the PB. In contrast, the proportions of T cell subtypes changed towards less naive Tconv and naive Treg in the PB, while the proportions of memory Tconv and memory Treg increased. Finally, expression of CD86 and CD80 costimulatory molecules on B cells as well as the expression of PD-1 on circulating T_FH_ were changed during fingolimod treatment.

We confirmed, as shown by others, that fingolimod reduced total lymphocyte, B and T cell numbers in the PB of MS patients [12], [23], [24]. For a comprehensive overview of the effects of fingolimod treatment on different cell types, we refer to the review of Brinkmann et al. [9].

The beneficial effects of fingolimod as MS treatment with minimal side effects could be attributed to different mechanisms of action. Fingolimod could entrap lymphocyte subtypes involved in MS pathogenesis in the lymph nodes by directly influencing migration of these lymphocytes from the lymph nodes into the circulation. As already reported by others, we show a decrease in peripheral memory B cells (both non class-switched and class-switched) while the naive B cell proportion increases [14], [15]. Although we do not report functional data, this finding could contribute to the beneficial effect of fingolimod treatment in MS patients. Memory B cells are largely implicated in MS pathogenesis as they are able to produce specific (auto)antibodies and are able to migrate to the CNS to enhance the ongoing immune response [3], [17], [25]. Entrapment of memory B cells in the lymph nodes could be a direct consequence of fingolimods' agonist activity on S1PR1 since egression of these cells could be mediated by S1PR1 signaling [14]. Additional proof of memory B cell entrapment in the lymph nodes comes from mice studies that showed a decrease in high-affinity class-switched antibodies by fingolimod, produced by memory B cells in the serum [26]. Furthermore, vaccination studies in fingolimod-treated healthy volunteers have demonstrated a mild to moderate decrease in immunoglobulin (Ig)G and IgM antibody levels towards some antigens [27].

B cells are important antigen presenting cells and recent evidence from mice studies has indicated that fingolimod can influence antigen handling [28]–[30]. In our study, B cell expression of the antigen presentation marker HLA-DR/DP/DQ and the percentage of HLA-DR/DP/DQ^+^ B cells was decreased in the PB during fingolimod treatment, which could be beneficial for MS pathogenesis since less antigen presentation occurs. However, this effect could also be attributed to a change in B cell subtype proportions in the PB.

Next to changes in B cells proportions, fingolimod treatment led to a decrease in the proportion of peripheral naive Tconv and an increase in the memory Tconv. Our results are in agreement with previous studies showing that effector memory T cells (TEM), lacking expression of C-C chemokine receptor type 7 (CCR7), were increased in the PB of fingolimod-treated MS patients [31]. It is thought that these circulating TEM have a suppressor function and downregulate the autoimmune response [10].

Furthermore, the homeostasis and function of Treg is disturbed in MS [32], [33]. In addition to the previously described increase in percentage of Tregs in the PB under fingolimod treatment, we show that the increase in Tregs is mostly attributed to an expansion in the memory population while a decrease in naive Treg cells was observed [13], [34]. Of note, an increase in memory Tregs could be responsible for recovery of Treg suppressive activity under fingolimod treatment as previously illustrated by our group for patients with SPMS [33]. It was already speculated that treatment with fingolimod works by both sequestering autoreactive B and T cells in the secondary lymphoid organs and by enhancing the functionality and frequency of circulating Treg [35].

As circulating T_FH_ are important for a normal germinal center response [36], we investigated whether these cells are affected by fingolimod treatment in the PB of MS patients and found that fingolimod treatment did not change the percentage of circulating T_FH_ cells. Recent evidence indicated that circulating T_FH_ cells consist of a CCR7^lo^PD-1^hi^ subpopulation with an effector phenotype. Therefore these cells could be less responsive to fingolimod as observed for memory Treg and Tconv cells [37].

Although fingolimod causes entrapment of lymphocytes in the lymph nodes, expression of surface molecules involved in costimulation was increased, which could point to a gain of functionality of the remaining circulating lymphocyte subtypes, although functional assessment is needed using both in vitro and animal studies to confirm this argument. During fingolimod treatment expression of CD86 and CD80 costimulatory molecules on B cells was increased and furthermore, an increase in PD-1 expression on T_FH_ cells was observed. The percentage of CD86^+^ B cells was increased as well during fingolimod treatment, which could be attributed to a change in B cell subtype distribution. Expression of CXCR5 on T_FH_ cells was unchanged during treatment, indicative of normal migration of these cells from the marginal zone to the follicles in the lymph nodes. Considering the beneficial clinical effects of fingolimod, we hypothesize that this increase in B and T cell costimulation and no change in migration capacity is a rescue mechanism to augment functionality of the remaining B and T cells, thereby warranting normal immunity. An additional proof of normal immune function is that vaccine specific production of IgM and IgG towards influenza A and B in fingolimod-treated individuals was not impaired when compared to levels in healthy controls [38].

Due to technical limitations and low cell numbers available for analysis, CD8^+^ and natural killer (NK) cells were not assessed. Further limitations of the study are the lack of functional data, although we provide evidence that during fingolimod treatment expression of functionally relevant markers on the remaining B and T cell subtypes in the peripheral blood of MS patients can change.

To conclude, this study shows that fingolimod induces compositional changes of B and T cell subtypes that are potentially implicated in MS pathogenesis and may explain the therapeutic efficacy of the treatment. While altered surface expression of functional molecules on B and T cells during fingolimod treatment suggests that normal immune function may prevail, functional evidence for this has still to be provided during future research. With this descriptive study we provide additional longitudinal immunological proof for the diverse mechanisms of action of fingolimod in MS patients.

## Supporting Information

Figure S1 **Proportional changes of B cell subtypes during fingolimod treatment in MS patients.** Proportional composition of (A) naive B cells, (B) non class-switched memory B cells, (C) class-switched memory B cells and (D) double negative B cells within the CD19+ B cell population of treatment-naive, IFN-β and fingolimod-treated MS patients.
(TIF)

Figure S2 **Compositional changes of T cell subtypes during fingolimod treatment in MS patients.** Proportional changes of (A) naive conventional T cells, (B) memory conventional T cells, (C) transitional conventional T cells, (D) naive regulatory T cells, (E) memory regulatory T cells and (F) transitional regulatory T cells. Changes are depicted as percentage within the CD4+ T cell population and measured in treatment-naive, IFN- β and fingolimod-treated MS patients.
(TIF)

Table S1 **Mean percentages of different B and T cell subtypes.**
(DOCX)

Table S2 **Mean fluorescence intensity and percentage positive cells of different surface markers on B and T cells.**
(DOCX)
