# Supplementary figures and images for: Compositional Changes of B and T Cell Subtypes during Fingolimod Treatment in Multiple Sclerosis Patients: A 12-Month Follow-Up Study
Source: PLoS One. 2014 Oct 31;9(10):e111115. doi: 10.1371/journal.pone.0111115 (PMC4215872; doi:10.1371/journal.pone.0111115)

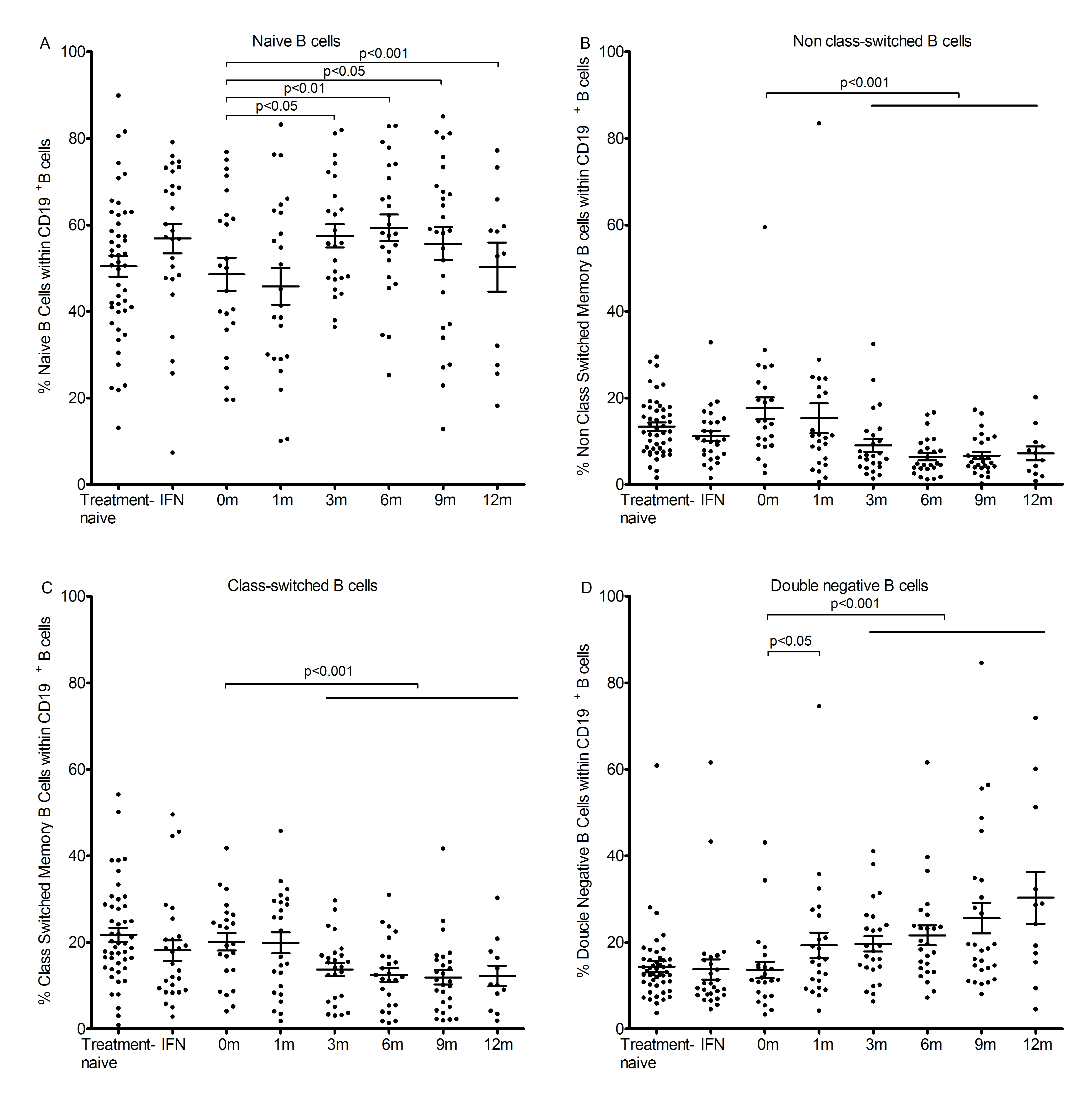

Supplement: Figure S1 — Proportional changes of B cell subtypes during fingolimod treatment in MS patients. Proportional composition of (A) naive B cells, (B) non class-switched memory B cells, (C) class-switched memory B cells and (D) double negative B cells within the CD19+ B cell population of treatment-naive, IFN-β and fingolimod-treated MS patients. (TIF) [file pone.0111115.s001.tif]

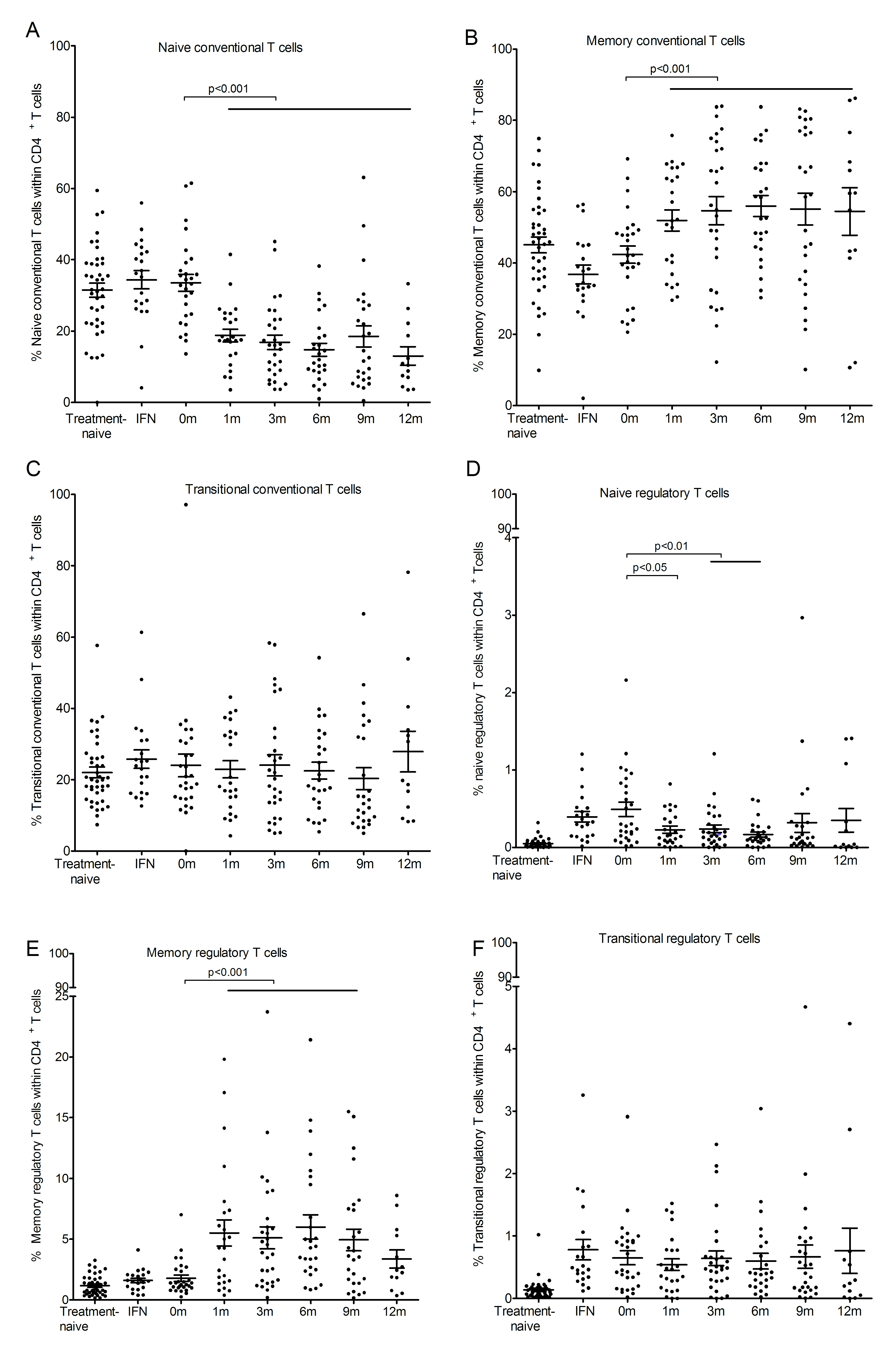

Supplement: Figure S2 — Compositional changes of T cell subtypes during fingolimod treatment in MS patients. Proportional changes of (A) naive conventional T cells, (B) memory conventional T cells, (C) transitional conventional T cells, (D) naive regulatory T cells, (E) memory regulatory T cells and (F) transitional regulatory T cells. Changes are depicted as percentage within the CD4+ T cell population and measured in treatment-naive, IFN- β and fingolimod-treated MS patients. (TIF) [file pone.0111115.s002.tif]
