# Supplementary material for: Compositional Changes of B and T Cell Subtypes during Fingolimod Treatment in Multiple Sclerosis Patients: A 12-Month Follow-Up Study
Source: PLoS One. 2014 Oct 31;9(10):e111115. doi: 10.1371/journal.pone.0111115 (PMC4215872; doi:10.1371/journal.pone.0111115)
Supplement: Table S1 — Mean percentages of different B and T cell subtypes. (DOCX) [file pone.0111115.s003.docx]

| *Table S1: Mean percentages of different B and T cell subtypes.* | | | | | | | | | | | | | | | | | | | | | | | | |
| --- | --- | --- | --- | --- | --- | --- | --- | --- | --- | --- | --- | --- | --- | --- | --- | --- | --- | --- | --- | --- | --- | --- | --- | --- |
| Subtype | Treatment naive | | | IFN-β | | | Fingolimod (Months) | | | | | | | | | | | | | | | | | |
|  |  |  |  |  |  |  | 0 | | | 1 | | | 3 | | | 6 | | | 9 | | | 12 | | |
|  | Mean | Sem | n | Mean | Sem | n | Mean | Sem | n | Mean | Sem | n | Mean | Sem | n | Mean | Sem | n | Mean | Sem | n | Mean | Sem | n |
| **CD4^+^** | 40.81 | 1.84 | 40 | 48.60 | 2.02 | 21 | 33.27 | 2.77 | 28 | **9.01¥** | **1.77** | **24** | **9.49¥** | **1.71** | **29** | **5.09¥** | **0.88** | **26** | **5.82¥** | **1.46** | **26** | **5.35¥** | **2.07** | **13** |
| Tconv | 98.60 | 0.14 | 40 | **96.95*** | **0.30** | **21** | **96.99*** | **0.33** | **28** | **93.62¥** | **1.15** | **24** | **93.83¥** | **0.91** | **29** | **93.27¥** | **0.98** | **26** | **93.94£** | **0.91** | **26** | **95.34** | **1.16** | **13** |
| mTconv | 45.08 | 2.24 | 40 | 36.77 | 2.64 | 21 | 42.34 | 2.42 | 27 | **51.93¥** | **2.96** | **24** | **54.67¥** | **3.95** | **28** | **55.97¥** | **2.93** | **26** | **55.12¥** | **4.44** | **26** | **54.42¥** | **6.69** | **13** |
| nTconv | 31.47 | 1.98 | 40 | 34.39 | 2.57 | 21 | 33.51 | 2.34 | 27 | **18.78¥** | **1.76** | **24** | **16.84¥** | **2.01** | **29** | **14.77¥** | **1.81** | **26** | **18.50¥** | **2.99** | **26** | **13.01¥** | **2.60** | **13** |
| Trans Tconv | 22.05 | 1.53 | 40 | 25.79 | 2.56 | 21 | 23.99 | 3.19 | 28 | 22.91 | 2.42 | 24 | 24.05 | 2.95 | 29 | 22.53 | 2.38 | 26 | 20.32 | 3.08 | 26 | 27.91 | 5.70 | 13 |
| T_FH_ | 2.52 | 0.37 | 37 | 2.22 | 0.34 | 20 | 2.92 | 0.59 | 28 | 2.59 | 0.46 | 24 | 2.99 | 0.89 | 29 | 2.76 | 0.66 | 26 | 2.80 | 0.75 | 26 | 2.54 | 1.17 | 13 |
| Treg | 1.37 | 0.14 | 40 | 2.79 | 0.27 | 21 | **2.94*** | **0.34** | **28** | **6.28¥** | **1.14** | **24** | **6.00¥** | **0.91** | **29** | **6.77¥** | **0.99** | **26** | **5.93£** | **0.93** | **26** | 4.49 | 1.14 | 13 |
| mTreg | 1.18 | 0.13 | 40 | 1.61 | 0.19 | 21 | 1.79 | 0.27 | 28 | **5.51¥** | **1.08** | **24** | **5.12¥** | **0.91** | **29** | **6.01¥** | **1.00** | **26** | **4.95¥** | **0.89** | **26** | 3.38 | 0.75 | 13 |
| nTreg | 0.05 | 0.01 | 40 | **0.40*** | **0.07** | **21** | **0.49*** | **0.09** | **28** | **0.23$** | **0.05** | **24** | **0.24£** | **0.05** | **29** | **0.17£** | **0.03** | **26** | 0.32 | 0.12 | 26 | 0.35 | 0.15 | 13 |
| M | 0.05 | 0.01 |  | **0.32*** | 0.05 |  | **0.81*** | 0.27 |  |  |  |  |  |  |  |  |  |  |  |  |  |  |  |  |
| F | 0.05 | 0.01 |  | **0.42*** | 0.09 |  | **0.37*** | 0.07 |  |  |  |  |  |  |  |  |  |  |  |  |  |  |  |  |
| Trans Treg | 0.14 | 0.03 | 40 | **0.78*** | 0.16 | 21 | **0.65*** | 0.11 | 28 | 0.54 | 0.01 | 24 | 0.65 | 0.12 | 29 | 0.60 | 0.13 | 26 | 0.67 | 0.19 | 26 | 0.77 | 0.36 | 13 |
| **CD19^+^** | 7.06 | 0.63 | 47 | 8.30 | 0.59 | 27 | 6.21 | 0.66 | 23 | **3.74¥** | **0.67** | **24** | **2.65¥** | **0.39** | **25** | **2.40¥** | **0.47** | **25** | **2.42¥** | **0.33** | **27** | **1.32¥** | **0.32** | **12** |
| Naive | 50.43 | 2.42 | 47 | 56.86 | 3.39 | 27 | 48.60 | 3.82 | 23 | 45.82 | 4.20 | 24 | **57.49$** | **2.65** | **25** | **59.35£** | **3.04** | **25** | **55.71$** | **3.80** | **27** | **50.25¥** | **5.66** | **12** |
| NCS memory | 13.39 | 0.96 | 47 | 11.24 | 1.23 | 27 | 17.63 | 2.53 | 23 | 15.36 | 3.42 | 24 | **9.05¥** | **1.48** | **25** | **6.49¥** | **0.88** | **25** | **6.70¥** | **0.85** | **27** | **7.21¥** | **1.62** | **12** |
| CS memory | 21.77 | 1.64 | 47 | 18.16 | 2.37 | 27 | 20.13 | 2.01 | 23 | 19.89 | 2.41 | 24 | **13.77¥** | **1.51** | **25** | **12.54¥** | **1.57** | **25** | **11.94¥** | **1.66** | **27** | **12.24¥** | **2.36** | **12** |
| Double negative | 14.41 | 1.24 | 47 | 13.78 | 2.32 | 27 | 13.67 | 1.90 | 23 | **19.35$** | **2.93** | **24** | **19.70¥** | **1.81** | **25** | **21.64¥** | **2.30** | **25** | **25.66¥** | **3.54** | **27** | **30.32¥** | **5.97** | **12** |
| * p < 0.05 versus treatment naive; # p < 0.05 versus interferon; $ p < 0.05 versus 0 months (SAS 9.3); £ p < 0.01 versus 0 months; ¥ p < 0.001 versus 0 months. Baseline fingolimod treatment was compared to treatment-naive and IFN-β-treated patients using appropriate one-way ANOVA (SAS 9.3). Interaction between treatment and sex was assessed and only observed for nTreg cells. Therefore, baseline differences were subdivided for males (M) and females (F). A mixed model was used for comparison of follow-up data with baseline. Abbreviations: Tconv = conventional T cells; mTconv = memory conventional T cells; nTconv = naive conventional T cells; Trans Tconv = transitional conventional T cells; T_FH_ = follicular helper T cells; Treg = regulatory T cells; mTreg = memory regulatory T cells; nTreg = naive regulatory T cells; trans Treg = transitional regulatory T cells; SEM = standard error of the mean; n = number of samples; NCS = non class-switched; CS = class-switched; M = male; F = female | | | | | | | | | | | | | | | | | | | | | | | | |
