# Supplementary material for: Compositional Changes of B and T Cell Subtypes during Fingolimod Treatment in Multiple Sclerosis Patients: A 12-Month Follow-Up Study
Source: PLoS One. 2014 Oct 31;9(10):e111115. doi: 10.1371/journal.pone.0111115 (PMC4215872; doi:10.1371/journal.pone.0111115)
Supplement: Table S2 — Mean fluorescence intensity and percentage positive cells of different surface markers on B and T cells. (DOCX) [file pone.0111115.s004.docx]

| Table S2: Mean fluorescence intensity and percentage positive cells of different surface markers on B and T cells. | | | | | | | | | | | | | | | | | | | | | | | | | |
| --- | --- | --- | --- | --- | --- | --- | --- | --- | --- | --- | --- | --- | --- | --- | --- | --- | --- | --- | --- | --- | --- | --- | --- | --- | --- |
| Subtype | Treatment naive | | | IFN-β | | | Fingolimod (Months) | | | | | | | | | | | | | | | | | |  |
|  |  |  |  |  |  |  | 0 | | | 1 | | | 3 | | | 6 | | | 9 | | | 12 | | |  |
|  | Mean | Sem | n | Mean | Sem | n | Mean | Sem | n | Mean | Sem | n | Mean | Sem | n | Mean | Sem | n | Mean | Sem | n | Mean | Sem | n |  |
| **MFI of T_FH_** |  |  |  |  |  |  |  |  |  |  |  |  |  |  |  |  |  |  |  |  |  |  |  |  |  |
| PD-1 | 3080 | 207.7 | 37 | 3634 | 425.7 | 20 | **2340#** | **65.50** | **26** | **3326$** | **192.7** | **22** | **3994$** | **384.1** | **28** | **3726$** | **272.2** | **27** | **3939$** | **289.7** | **24** | **4660¥** | **289.7** | **10** |  |
| CXCR5 | 2669 | 328.7 | 37 | 2001 | 328.4 | 20 | 1644 | 136.7 | 25 | 2126 | 234.7 | 22 | 2053 | 340.5 | 27 | 2770 | 441.2 | 25 | 2081 | 335.3 | 23 | 3847 | 919.0 | 12 |  |
| **% of CD19^+^** |  |  |  |  |  |  |  |  |  |  |  |  |  |  |  |  |  |  |  |  |  |  |  |  |  |
| MHC II | 98.30 | 0.294 | 47 | 98.36 | 0.391 | 27 | 98.37 | 0.34 | 23 | 97.64 | 0.44 | 24 | **95.32$** | 1.08 | 25 | **94.46$** | 1.93 | 25 | **92.69$** | 1.55 | 27 | **87.00$** | 4.74 | 12 |  |
| CD80 | 17.90 | 1.889 | 47 | 17.59 | 2.412 | 27 | 19.91 | 2.40 | 23 | 20.28 | 2.56 | 24 | 18.20 | 2.06 | 25 | 19.73 | 2.11 | 25 | 16.07 | 2.06 | 27 | 22.12 | 3.20 | 12 |  |
| CD86 | 15.31 | 2.034 | 47 | 17.60 | 2.178 | 27 | 17.03 | 2.78 | 23 | 19.61 | 2.77 | 24 | **23.65$** | 2.70 | 25 | 18.42 | 2.12 | 25 | 20.27 | 2.08 | 27 | **29.16$** | 4.42 | 12 |  |
| **MFI of CD19^+^** |  |  |  |  |  |  |  |  |  |  |  |  |  |  |  |  |  |  |  |  |  |  |  |  |  |
| MHC II | 17393 | 1143 | 47 | 16829 | 1742 | 27 | 20691 | 1978 | 23 | **16837$** | **1290** | **24** | **17453$** | **1557** | **25** | **17228£** | **1551** | **25** | **16304£** | **1400** | **27** | 19059 | 2053 | 12 |  |
| CD80 | 501.4 | 48.54 | 47 | 477.7 | 44.78 | 27 | 524.4 | 52.83 | 23 | 694.9 | 100.6 | 24 | **938.6$** | **205.2** | **25** | 730.2 | 67.82 | 25 | 638.6 | 131.4 | 27 | **1013$** | **173.9** | **12** |  |
| CD86 | 474.0 | 47.26 | 47 | 566.7 | 62.00 | 27 | 569.1 | 103.9 | 23 | **853.5$** | **156.5** | **24** | **1065£** | **125.5** | **25** | **864.4£** | **95.14** | **25** | **946.8$** | **197.7** | **27** | **1386£** | **320.3** | **12** |  |
| * p < 0.05 versus treatment naive; # p < 0.05 versus interferon; $ p < 0.05 versus 0 months; £ p < 0.01 versus 0 months; ¥ p < 0.001 versus 0 months  Abbreviations: PD-1 = programmed cell death 1; MFI = mean fluorescence intensity; CXCR5 = CXC motif receptor 5; TFH = follicular helper cells; MHC II = major histocompatibility II = HLA-DR/DP/DQ; CD = cluster of differentiation; Sem = standard error of the mean; n = number of samples | | | | | | | | | | | | | | | | | | | | | | | | |  |
